# Corymbolone

**DOI:** 10.1107/S1600536814012938

**Published:** 2014-06-11

**Authors:** Stacey Burrett, Dennis K. Taylor, Edward R. T. Tiekink

**Affiliations:** a School of Agriculture, Food and Wine, The University of Adelaide, Waite Campus, PMB 1, Glen Osmond, SA 5064, Australia; b Department of Chemistry, University of Malaya, 50603 Kuala Lumpur, Malaysia

## Abstract

The title compound, C_15_H_24_O_2_ [systematic name: (4*S*,4a*R*,6*R*,8a*R*)-4a-hy­droxy-4,8a-dimethyl-6-(prop-1-en-2-yl)octahydro­naphthalen-1(2*H*)-one], features two edge-shared six-membered rings with the hydroxyl and methyl substituents at this bridge being *trans*. One adopts a flattened chair conformation with the C atoms bearing the carbonyl and methyl substituents lying 0.5227 (16) and 0.6621 (15) Å, respectively, above and below the mean plane through the remaining four C atoms (r.m.s. deviation = 0.0145 Å). The second ring, bearing the prop-1-en-2-yl group, has a chair conformation. Supra­molecular helical chains along the *b* axis are found in the crystal packing, which are sustained by hy­droxy–carbonyl O—H⋯O hydrogen bonding.

## Related literature   

For the first isolation and the spectroscopic data of corymbolone, see: Garbarino *et al.* (1985 ▶). For the synthesis of corymbolone in racemic form, see: Ferraz *et al.* (2006 ▶).
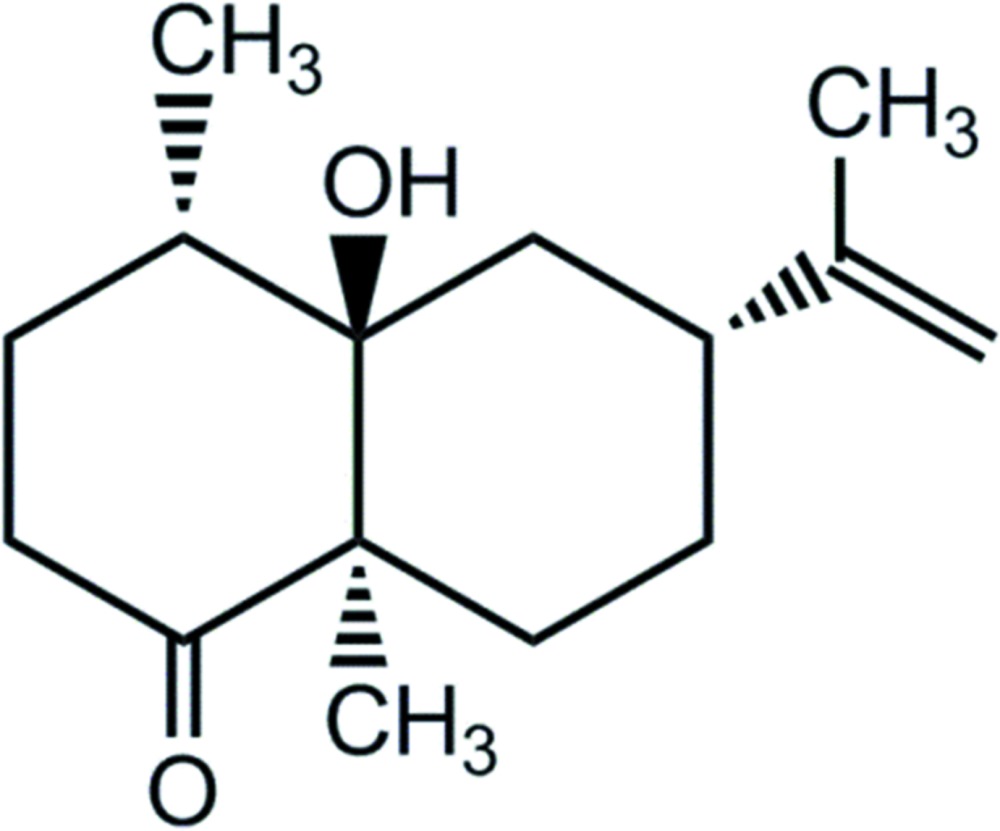


## Experimental   

### 

#### Crystal data  

C_15_H_24_O_2_
*M* *_r_* = 236.34
Monoclinic, 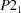

*a* = 6.1057 (2) Å
*b* = 12.1389 (2) Å
*c* = 9.2737 (2) Å
β = 99.302 (2)°
*V* = 678.30 (3) Å^3^
*Z* = 2
Cu *K*α radiation
μ = 0.58 mm^−1^
*T* = 100 K
0.30 × 0.25 × 0.20 mm

#### Data collection  

Agilent SuperNova Dual diffractometer with an Atlas detector
Absorption correction: multi-scan (*CrysAlis PRO*; Agilent, 2013 ▶) *T*_min_ = 0.689, *T*_max_ = 1.000
4848 measured reflections
2631 independent reflections
2621 reflections with *I* > 2σ(*I*)
*R*_int_ = 0.011

#### Refinement  

*R*[*F* ^2^ > 2σ(*F* ^2^)] = 0.030
*wR*(*F* ^2^) = 0.083
*S* = 1.03
2631 reflections
161 parameters
1 restraint
H atoms treated by a mixture of independent and constrained refinement
Δρ_max_ = 0.24 e Å^−3^
Δρ_min_ = −0.14 e Å^−3^
Absolute structure: Flack (1983 ▶), 1200 Friedel pairs
Absolute structure parameter: 0.02 (16)

### 

Data collection: *CrysAlis PRO* (Agilent, 2013 ▶); cell refinement: *CrysAlis PRO*; data reduction: *CrysAlis PRO*; program(s) used to solve structure: *SHELXS97* (Sheldrick, 2008 ▶); program(s) used to refine structure: *SHELXL97* (Sheldrick, 2008 ▶); molecular graphics: *ORTEP-3 for Windows* (Farrugia, 2012 ▶) and *DIAMOND* (Brandenburg, 2006 ▶); software used to prepare material for publication: *publCIF* (Westrip, 2010 ▶).

## Supplementary Material

Crystal structure: contains datablock(s) general, I. DOI: 10.1107/S1600536814012938/su2741sup1.cif

Structure factors: contains datablock(s) I. DOI: 10.1107/S1600536814012938/su2741Isup2.hkl

Supporting information file. DOI: 10.1107/S1600536814012938/su2741Isup3.cml

CCDC reference: 1006604

Additional supporting information:  crystallographic information; 3D view; checkCIF report

## Figures and Tables

**Table 1:** Hydrogen-bond geometry (Å, °)

*D*—H⋯*A*	*D*—H	H⋯*A*	*D*⋯*A*	*D*—H⋯*A*
O2—H2⋯O1^i^	0.863 (19)	1.993 (19)	2.8513 (12)	172.5 (16)

Symmetry code: (i) 
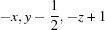
.

**Fig. 1.**
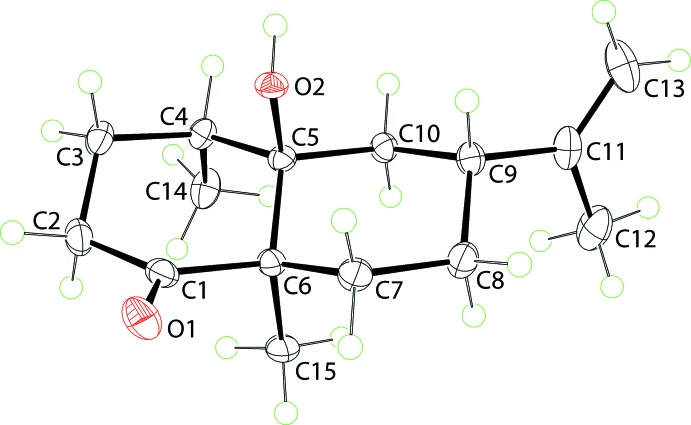
The molecular structure of the title molecule, with atom labelling. Displacement ellipsoids are drawn at the 50% probability level.

**Fig. 2.**
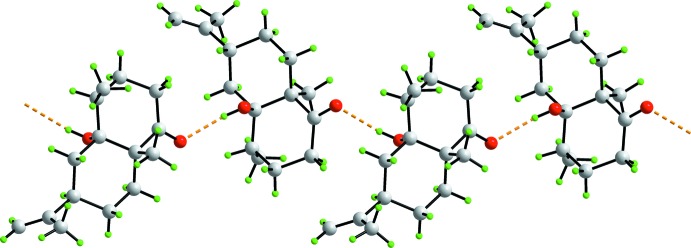
A view of the helical supramolecular chain along the b axis in the title compound. The O—H···O hydrogen bonds are shown as orange dashed lines.

## supplementary crystallographic information

### S1. Structural commentary

The title compound, corymbolone, was first characterised in 1985 (Garbarino *et al.*, 1985), and more recently synthesized in racemic form (Ferraz *et al.*, 2006). In the present study, it was isolated from the product mixture that resulted from aerial oxidation of α-guaiene.

The molecular structure of the title molecule, Fig. 1, features two fused six-membered rings. The C2,C3,C5 and C6 atoms of the C1–C6 ring are planar with a r.m.s. deviation of 0.0145 Å, and with the C1 and C4 atoms lying 0.5227 (16) and 0.6621 (15) Å above and below this plane, respectively, so that the conformation of the ring is best described as being a flattened chair. By contrast, the C5–C10 ring closely approximates a chair conformation. With respect to the C1–C6 ring the C1-carbonyl, C4-methyl, C5-hydroxyl and C6-methyl groups have equatorial (eq), axial (ax), ax and eq dispositions, respectively. For the C5–C10 ring, the C5-hydroxyl, C6-methyl and C9-prop-1-en-2-yl groups have have ax, ax and eq dispositions, respectively.

The most prominent feature of the crystal packing is the formation of hydroxyl-O—H···O(carbonyl) hydrogen bonding that leads to helical supra­molecular chains along the *b* axis (Table 1 and Fig. 2).

### S2. Synthesis and crystallization

Air was slowly bubbled through a neat solution of α-guaiene (7.0 g, 34.3 mmol) and after 21 days the crude mixture of products was subjected to column chromatography with a gradient of 100% hexane to 100% EtOAc. The product (0.12 g, 1.5%) at Rf 0.07 (10% EtOAc/hexane) was collected as a white crystalline solid and recrystallized from hexane to afford block-like colourless crystals of corymboline. M.p. 408–409 K; Lit. M.p. 409–410 K (Garbarino *et al.*, 1985). Spectroscopic data for the title compound are available in the archived CIF.

### S3. Refinement

The hy­droxy-H atom was located in a difference Fourier map and freely refined. C-bound H-atoms were placed in calculated positions [C—H = 0.95 - 1.00 Å] and included in the refinement in the riding model approximation with *U*_iso_(H) = 1.5*U*_eq_(C-methyl) and = 1.2U_eq_(C) for other H atoms. Owing to poor agreement, two reflections, *i.e*. (1 1 0) and (2 -4 2), were omitted from the final cycles of refinement.

### Crystal data

**Table:**

C_15_H_24_O_2_	*F*(000) = 260
*M**_r_* = 236.34	*D*_x_ = 1.157 Mg m^−^^3^
Monoclinic, *P*2_1_	Cu *K*α radiation, λ = 1.54184 Å
Hall symbol: P 2yb	Cell parameters from 3768 reflections
*a* = 6.1057 (2) Å	θ = 3.6–74.3°
*b* = 12.1389 (2) Å	µ = 0.58 mm^−^^1^
*c* = 9.2737 (2) Å	*T* = 100 K
β = 99.302 (2)°	Block, colourless
*V* = 678.30 (3) Å^3^	0.30 × 0.25 × 0.20 mm
*Z* = 2	

### Data collection

**Table:**

Agilent SuperNova Dual diffractometer with an Atlas detector	2631 independent reflections
Radiation source: SuperNova (Cu) X-ray Source	2621 reflections with *I* > 2σ(*I*)
Mirror monochromator	*R*_int_ = 0.011
Detector resolution: 10.4041 pixels mm^-1^	θ_max_ = 74.5°, θ_min_ = 6.1°
ω scan	*h* = −7→7
Absorption correction: multi-scan (*CrysAlis PRO*; Agilent, 2013)	*k* = −14→14
*T*_min_ = 0.689, *T*_max_ = 1.000	*l* = −11→11
4848 measured reflections	

### Refinement

**Table:**

Refinement on *F*^2^	Secondary atom site location: difference Fourier map
Least-squares matrix: full	Hydrogen site location: inferred from neighbouring sites
*R*[*F*^2^ > 2σ(*F*^2^)] = 0.030	H atoms treated by a mixture of independent and constrained refinement
*wR*(*F*^2^) = 0.083	*w* = 1/[σ^2^(*F*_o_^2^) + (0.0567*P*)^2^ + 0.0924*P*] where *P* = (*F*_o_^2^ + 2*F*_c_^2^)/3
*S* = 1.03	(Δ/σ)_max_ < 0.001
2631 reflections	Δρ_max_ = 0.24 e Å^−^^3^
161 parameters	Δρ_min_ = −0.14 e Å^−^^3^
1 restraint	Absolute structure: Flack (1983), 1200 Friedel pairs
Primary atom site location: structure-invariant direct methods	Absolute structure parameter: 0.02 (16)

### Special details

**Table:**

Experimental. Spectroscopic data for the title compound, ^1^H NMR (600 MHz, CDCl_3_) δ 4.74 (s, 2H), 2.68 (ddd, J = 17.2, 9.9, 7.8 Hz, 1H), 2.44-2.36 (m, 2H), 2.32 (dddd, J = 12.0, 12.0, 4.2, 4.2 Hz, 1H), 1.93-1.83 (m, 3H), 1.75 (s, 3H), 1.71-1.65 (m, 2H), 1.60 (ddd, J = 13.8, 3.0, 3.0 Hz, 1H), 1.43 (ddd, J = 13.7, 3.7, 2.0 Hz, 1H), 1.37 (dddd, J = 13.3, 13.3, 13.3, 3.6 Hz, 1H), 1.29 (br, 1H), 1.24 (s, 3H), 1.19 (d, J = 7.8 Hz, 3H); ^13^C NMR (600 MHz, CDCl_3_) δ 215.8, 149.5, 108.9, 78.6, 51.2, 40.6, 39.4, 37.2, 34.2, 30.2, 28.0, 25.5, 21.1, 20.4, 17.8; MS: m/z (%) 236 (8), 218 (17), 203 (33), 175 (28), 153 (27), 137 (35), 135 (42), 124 (40), 109 (100), 93 (50), 84 (27), 69 (57), 55 (62), 41 (67). All other physical and spectral data were identical to those previously reported by Garbarino *et al.* (1985).
Geometry. All e.s.d.'s (except the e.s.d. in the dihedral angle between two l.s. planes) are estimated using the full covariance matrix. The cell e.s.d.'s are taken into account individually in the estimation of e.s.d.'s in distances, angles and torsion angles; correlations between e.s.d.'s in cell parameters are only used when they are defined by crystal symmetry. An approximate (isotropic) treatment of cell e.s.d.'s is used for estimating e.s.d.'s involving l.s. planes.
Refinement. Refinement of *F*^2^ against ALL reflections. The weighted *R*-factor *wR* and goodness of fit *S* are based on *F*^2^, conventional *R*-factors *R* are based on *F*, with *F* set to zero for negative *F*^2^. The threshold expression of *F*^2^ > σ(*F*^2^) is used only for calculating *R*-factors(gt) *etc*. and is not relevant to the choice of reflections for refinement. *R*-factors based on *F*^2^ are statistically about twice as large as those based on *F*, and *R*- factors based on ALL data will be even larger.

### Fractional atomic coordinates and isotropic or equivalent isotropic displacement parameters (Å^2^)

**Table:**

	*x*	*y*	*z*	*U*_iso_*/*U*_eq_	
O1	−0.00899 (15)	0.49702 (8)	0.42187 (10)	0.0276 (2)	
O2	0.02823 (12)	0.20248 (7)	0.43171 (9)	0.01677 (18)	
H2	0.026 (3)	0.1374 (16)	0.4692 (19)	0.025 (4)*	
C1	0.1255 (2)	0.42790 (10)	0.47357 (14)	0.0195 (2)	
C2	0.2007 (2)	0.41919 (11)	0.63667 (14)	0.0218 (3)	
H2A	0.0916	0.4578	0.6868	0.026*	
H2B	0.3444	0.4579	0.6620	0.026*	
C3	0.2279 (2)	0.30153 (11)	0.69525 (13)	0.0204 (3)	
H3A	0.0793	0.2687	0.6946	0.024*	
H3B	0.3047	0.3035	0.7978	0.024*	
C4	0.36071 (18)	0.22809 (10)	0.60493 (12)	0.0177 (2)	
H4	0.3363	0.1506	0.6352	0.021*	
C5	0.25743 (17)	0.23588 (9)	0.44117 (12)	0.0145 (2)	
C6	0.24052 (19)	0.35347 (10)	0.37568 (13)	0.0162 (2)	
C7	0.1070 (2)	0.34852 (10)	0.22026 (13)	0.0201 (2)	
H7A	−0.0456	0.3229	0.2250	0.024*	
H7B	0.0969	0.4233	0.1772	0.024*	
C8	0.2149 (2)	0.27089 (11)	0.12238 (13)	0.0212 (3)	
H8A	0.3607	0.3012	0.1089	0.025*	
H8B	0.1204	0.2668	0.0251	0.025*	
C9	0.2478 (2)	0.15434 (10)	0.18670 (13)	0.0179 (2)	
H9	0.0971	0.1229	0.1891	0.021*	
C10	0.37049 (19)	0.15834 (9)	0.34538 (13)	0.0165 (2)	
H10A	0.3764	0.0832	0.3874	0.020*	
H10B	0.5249	0.1833	0.3457	0.020*	
C11	0.3657 (2)	0.07717 (11)	0.09544 (13)	0.0234 (3)	
C12	0.5839 (2)	0.11391 (13)	0.05605 (16)	0.0319 (3)	
H12A	0.6510	0.0530	0.0093	0.048*	
H12B	0.6837	0.1363	0.1448	0.048*	
H12C	0.5591	0.1764	−0.0116	0.048*	
C13	0.2792 (3)	−0.02085 (13)	0.05543 (16)	0.0351 (3)	
H13A	0.3555	−0.0696	0.0006	0.042*	
H13B	0.1412	−0.0421	0.0817	0.042*	
C14	0.6114 (2)	0.24905 (12)	0.64454 (14)	0.0249 (3)	
H14A	0.6881	0.2150	0.5709	0.037*	
H14B	0.6664	0.2170	0.7406	0.037*	
H14C	0.6396	0.3286	0.6474	0.037*	
C15	0.4677 (2)	0.40859 (11)	0.36941 (14)	0.0234 (3)	
H15A	0.5606	0.3583	0.3228	0.035*	
H15B	0.5415	0.4254	0.4688	0.035*	
H15C	0.4445	0.4769	0.3127	0.035*	

### Atomic displacement parameters (Å^2^)

**Table:**

	*U*^11^	*U*^22^	*U*^33^	*U*^12^	*U*^13^	*U*^23^
O1	0.0305 (5)	0.0183 (5)	0.0343 (5)	0.0084 (4)	0.0058 (4)	−0.0029 (4)
O2	0.0142 (4)	0.0150 (4)	0.0219 (4)	−0.0027 (3)	0.0056 (3)	0.0001 (3)
C1	0.0194 (5)	0.0139 (6)	0.0266 (6)	−0.0026 (4)	0.0078 (4)	−0.0024 (5)
C2	0.0211 (6)	0.0228 (6)	0.0230 (6)	−0.0009 (5)	0.0076 (4)	−0.0077 (5)
C3	0.0181 (5)	0.0269 (7)	0.0173 (6)	−0.0012 (5)	0.0066 (4)	−0.0028 (5)
C4	0.0166 (5)	0.0205 (6)	0.0169 (5)	0.0016 (4)	0.0051 (4)	0.0001 (4)
C5	0.0124 (5)	0.0145 (5)	0.0173 (5)	−0.0005 (4)	0.0047 (4)	0.0001 (4)
C6	0.0172 (5)	0.0135 (5)	0.0191 (5)	−0.0007 (4)	0.0068 (4)	−0.0011 (4)
C7	0.0244 (6)	0.0159 (6)	0.0203 (5)	0.0050 (5)	0.0044 (4)	0.0032 (5)
C8	0.0254 (6)	0.0223 (6)	0.0165 (5)	0.0034 (5)	0.0050 (4)	0.0009 (5)
C9	0.0173 (5)	0.0184 (6)	0.0181 (5)	0.0024 (4)	0.0037 (4)	−0.0024 (4)
C10	0.0174 (5)	0.0156 (5)	0.0172 (5)	0.0023 (4)	0.0046 (4)	−0.0008 (4)
C11	0.0235 (6)	0.0291 (7)	0.0168 (6)	0.0087 (5)	0.0011 (5)	−0.0039 (5)
C12	0.0321 (7)	0.0383 (8)	0.0285 (6)	0.0116 (6)	0.0143 (5)	0.0000 (6)
C13	0.0343 (7)	0.0341 (8)	0.0352 (7)	0.0082 (6)	0.0006 (6)	−0.0173 (6)
C14	0.0169 (5)	0.0379 (8)	0.0198 (6)	0.0035 (5)	0.0024 (4)	−0.0035 (5)
C15	0.0251 (6)	0.0190 (6)	0.0286 (6)	−0.0078 (5)	0.0125 (5)	−0.0024 (5)

### Geometric parameters (Å, º)

**Table:**

O1—C1	1.2171 (16)	C8—C9	1.5360 (17)
O2—C5	1.4459 (12)	C8—H8A	0.9900
O2—H2	0.863 (19)	C8—H8B	0.9900
C1—C2	1.5117 (17)	C9—C11	1.5193 (16)
C1—C6	1.5294 (15)	C9—C10	1.5403 (15)
C2—C3	1.5277 (19)	C9—H9	1.0000
C2—H2A	0.9900	C10—H10A	0.9900
C2—H2B	0.9900	C10—H10B	0.9900
C3—C4	1.5405 (15)	C11—C13	1.330 (2)
C3—H3A	0.9900	C11—C12	1.505 (2)
C3—H3B	0.9900	C12—H12A	0.9800
C4—C14	1.5365 (16)	C12—H12B	0.9800
C4—C5	1.5503 (15)	C12—H12C	0.9800
C4—H4	1.0000	C13—H13A	0.9500
C5—C10	1.5329 (15)	C13—H13B	0.9500
C5—C6	1.5482 (16)	C14—H14A	0.9800
C6—C7	1.5380 (16)	C14—H14B	0.9800
C6—C15	1.5493 (16)	C14—H14C	0.9800
C7—C8	1.5293 (17)	C15—H15A	0.9800
C7—H7A	0.9900	C15—H15B	0.9800
C7—H7B	0.9900	C15—H15C	0.9800
			
C5—O2—H2	108.0 (11)	C7—C8—H8A	109.2
O1—C1—C2	121.25 (11)	C9—C8—H8A	109.2
O1—C1—C6	121.25 (11)	C7—C8—H8B	109.2
C2—C1—C6	117.28 (10)	C9—C8—H8B	109.2
C1—C2—C3	114.78 (10)	H8A—C8—H8B	107.9
C1—C2—H2A	108.6	C11—C9—C8	113.31 (10)
C3—C2—H2A	108.6	C11—C9—C10	110.53 (9)
C1—C2—H2B	108.6	C8—C9—C10	110.78 (9)
C3—C2—H2B	108.6	C11—C9—H9	107.3
H2A—C2—H2B	107.5	C8—C9—H9	107.3
C2—C3—C4	112.60 (9)	C10—C9—H9	107.3
C2—C3—H3A	109.1	C5—C10—C9	112.21 (9)
C4—C3—H3A	109.1	C5—C10—H10A	109.2
C2—C3—H3B	109.1	C9—C10—H10A	109.2
C4—C3—H3B	109.1	C5—C10—H10B	109.2
H3A—C3—H3B	107.8	C9—C10—H10B	109.2
C14—C4—C3	111.45 (10)	H10A—C10—H10B	107.9
C14—C4—C5	117.12 (9)	C13—C11—C12	121.63 (13)
C3—C4—C5	109.32 (9)	C13—C11—C9	120.31 (13)
C14—C4—H4	106.1	C12—C11—C9	118.04 (12)
C3—C4—H4	106.1	C11—C12—H12A	109.5
C5—C4—H4	106.1	C11—C12—H12B	109.5
O2—C5—C10	108.35 (9)	H12A—C12—H12B	109.5
O2—C5—C6	103.43 (8)	C11—C12—H12C	109.5
C10—C5—C6	110.27 (9)	H12A—C12—H12C	109.5
O2—C5—C4	106.19 (8)	H12B—C12—H12C	109.5
C10—C5—C4	112.35 (9)	C11—C13—H13A	120.0
C6—C5—C4	115.55 (9)	C11—C13—H13B	120.0
C1—C6—C7	110.78 (10)	H13A—C13—H13B	120.0
C1—C6—C5	108.65 (9)	C4—C14—H14A	109.5
C7—C6—C5	108.86 (10)	C4—C14—H14B	109.5
C1—C6—C15	105.47 (10)	H14A—C14—H14B	109.5
C7—C6—C15	108.88 (9)	C4—C14—H14C	109.5
C5—C6—C15	114.18 (9)	H14A—C14—H14C	109.5
C8—C7—C6	111.50 (9)	H14B—C14—H14C	109.5
C8—C7—H7A	109.3	C6—C15—H15A	109.5
C6—C7—H7A	109.3	C6—C15—H15B	109.5
C8—C7—H7B	109.3	H15A—C15—H15B	109.5
C6—C7—H7B	109.3	C6—C15—H15C	109.5
H7A—C7—H7B	108.0	H15A—C15—H15C	109.5
C7—C8—C9	112.27 (9)	H15B—C15—H15C	109.5
			
O1—C1—C2—C3	140.04 (12)	C10—C5—C6—C7	58.86 (11)
C6—C1—C2—C3	−45.32 (15)	C4—C5—C6—C7	−172.39 (9)
C1—C2—C3—C4	47.77 (14)	O2—C5—C6—C15	−178.70 (9)
C2—C3—C4—C14	79.04 (13)	C10—C5—C6—C15	−63.02 (12)
C2—C3—C4—C5	−52.02 (12)	C4—C5—C6—C15	65.72 (12)
C14—C4—C5—O2	174.50 (10)	C1—C6—C7—C8	−177.79 (10)
C3—C4—C5—O2	−57.55 (11)	C5—C6—C7—C8	−58.38 (12)
C14—C4—C5—C10	56.22 (14)	C15—C6—C7—C8	66.66 (13)
C3—C4—C5—C10	−175.83 (9)	C6—C7—C8—C9	55.87 (13)
C14—C4—C5—C6	−71.50 (13)	C7—C8—C9—C11	−176.99 (10)
C3—C4—C5—C6	56.45 (11)	C7—C8—C9—C10	−52.10 (13)
O1—C1—C6—C7	−20.66 (16)	O2—C5—C10—C9	55.14 (12)
C2—C1—C6—C7	164.70 (10)	C6—C5—C10—C9	−57.40 (12)
O1—C1—C6—C5	−140.19 (11)	C4—C5—C10—C9	172.14 (9)
C2—C1—C6—C5	45.17 (13)	C11—C9—C10—C5	179.72 (10)
O1—C1—C6—C15	96.99 (13)	C8—C9—C10—C5	53.27 (13)
C2—C1—C6—C15	−77.64 (13)	C8—C9—C11—C13	−128.63 (13)
O2—C5—C6—C1	63.91 (10)	C10—C9—C11—C13	106.35 (14)
C10—C5—C6—C1	179.59 (9)	C8—C9—C11—C12	53.00 (15)
C4—C5—C6—C1	−51.67 (11)	C10—C9—C11—C12	−72.02 (14)
O2—C5—C6—C7	−56.81 (10)		

### Hydrogen-bond geometry (Å, º)

**Table:**

*D*—H···*A*	*D*—H	H···*A*	*D*···*A*	*D*—H···*A*
O2—H2···O1^i^	0.863 (19)	1.993 (19)	2.8513 (12)	172.5 (16)

Symmetry code: (i) −*x*, *y*−1/2, −*z*+1.
